# Growth/differentiation factor 15 (GDF15) expression in the heart after myocardial infarction and cardioprotective effect of pre-ischemic rGDF15 administration

**DOI:** 10.1038/s41598-024-63880-5

**Published:** 2024-06-05

**Authors:** Geoffrey Dogon, Eve Rigal, Eliot Potel, Marie Josse, Luc Rochette, Yannick Bejot, Catherine Vergely

**Affiliations:** 1 Research Team: Physiopathologie et Epidémiologie Cérébro-Cardiovasculaires (PEC2), Faculty of Health Sciences, University of Burgundy, 7 Bd Jeanne d’Arc, 21000 Dijon, France; 2 Department of Neurology, Dijon University Hospital, Dijon, France

**Keywords:** GDF-15, Ischemia–reperfusion injury, Heart, Preconditioning, Cardioprotection, Cardiovascular biology, Cardiology

## Abstract

Growth/differentiation factor-15 (GDF15) is considered an unfavourable prognostic biomarker for cardiovascular disease in clinical data, while experimental studies suggest it has cardioprotective potential. This study focuses on the direct cardiac effects of GDF15 during ischemia–reperfusion injury in Wistar male rats, employing concentrations relevant to patients at high cardiovascular risk. Initially, we examined circulating levels and heart tissue expression of GDF15 in rats subjected to ischemia–reperfusion and sham operations in vivo. We then evaluated the cardiac effects of GDF15 both in vivo and ex vivo, administering recombinant GDF15 either before 30 min of ischemia (preconditioning) or at the onset of reperfusion (postconditioning). We compared infarct size and cardiac contractile recovery between control and rGDF15-treated rats. Contrary to our expectations, ischemia–reperfusion did not increase GDF15 plasma levels compared to sham-operated rats. However, cardiac protein and mRNA expression increased in the infarcted zone of the ischemic heart after 24 h of reperfusion. Notably, preconditioning with rGDF15 had a cardioprotective effect, reducing infarct size both in vivo (65 ± 5% in control vs. 42 ± 6% in rGDF15 groups) and ex vivo (60 ± 4% in control vs. 45 ± 4% in rGDF15 groups), while enhancing cardiac contractile recovery ex vivo. However, postconditioning with rGDF15 did not alter infarct size or the recovery of contractile parameters in vivo or ex vivo. These novel findings reveal that the short-term exogenous administration of rGDF15 before ischemia, at physiologically relevant levels, protects the heart against ischemia–reperfusion injury in both in vivo and ex vivo settings. The ex vivo results indicate that rGDF15 operates independently of the inflammatory, endocrine and nervous systems, suggesting direct and potent cardioprotective properties against ischemia–reperfusion injury.

## Introduction

Growth/differentiation factor 15 (GDF15) stands out as a distinct member of the transforming growth factor superfamily (TGF-β)^1^. Initially produced as a ≈ 40 kDa pro-peptide monomer, it undergoes processing into a biologically active mature homodimer of ≈ 25 kDa. After secretion, the mature form of GDF15 can either be released or stored within the matrix^2^. Contrary to traditional TGF-β members, recent research highlights that GDF15 does not bind to TGF-β receptors. Instead, it interacts with the endogenous receptor GDNF-family α-like (GFRAL), which is exclusively present in the hindbrain^3–6^. Activation of GFRAL by GDF15 is involved in the regulation of metabolism, notably influencing appetite and regulating body mass, further emphasizing its uniqueness within the TGF-β superfamily^7^. This distinctive profile suggests that GDF15 might be more appropriately classified within the GDNF family.

GDF15 plays diverse and significant roles in various pathophysiological conditions, including cancer, inflammation, and cardiovascular disease^8,9^. It is emerging as a important biomarker^10^, particularly for identifying patients at risk of a poor prognosis^11–13^. While its expression is typically low in healthy tissues, including in the heart, pathological conditions such as cardiovascular (CV) comorbidities, oxidative stress, or hypoxia significantly increase its expression^14^. Notably, patients with atrial fibrillation^15,16^, heart failure^17,18^, atherosclerosis^19^, coronary artery diseases (CAD)^20^ and myocardial infarction (MI)^21^ exhibit high levels of GDF15. Unlike some other cardiovascular biomarkers, GDF15 plasma levels remain remarkably stable in CAD patients, both in the acute phase of coronary syndrome and in stable CAD, providing valuable short- to long-term prognostic information^22^. Patients with acute coronary syndrome and elevated GDF15 concentrations (> 1800 ng/L) face an increased risk of all-cause mortality, CV mortality and MI^23^, even after adjusting for classic biomarkers such as hs-troponin T, cystatin C, hs-CRP and NT-proBNP^20,24^. While GDF15 is expressed in the infarcted myocardium, the correlation between GDF15 and myocardial damage or function is still debated^25–27^.

Experimental research from our team has revealed an elevation in GDF15 levels in the blood after MI and ischemic stroke, accompanied by increased GDF15 pro-peptide expression in the surrounding ischemic area^28^. In vitro studies have demonstrated that cardiomyocytes produce GDF15 under hypoxia and in response to other stressors such as pro-inflammatory cytokines, oxidative stress, and mechanical stretch^25,29^. Intriguingly, GDF15 exhibits cardioprotective properties by reducing cardiomyocyte apoptosis during simulated ischemia–reperfusion (I/R) through activation of the PI3K-Akt signalling pathway^30^, although no cardiac GDF15 receptor has been identified to date. In vivo studies in GDF15-deficient mice revealed increased infarct sizes and more apoptotic cardiomyocytes after I/R^25,31^. These effects are thought to be mediated through direct cardiomyocyte protection^25^ and by regulating inflammation, modulating leukocyte infiltration in the infarcted area^32^ and promoting immune tolerance^33^.

This study initially sought to analyse the expression and secretion kinetics of GDF15 in response to myocardial I/R. We also investigated its direct cardiac effects in both in vivo and ex vivo models of I/R using recombinant GDF15 concentrations relevant to patients with a high CV risk, to assess the potential suitability of rGDF15 as a therapeutic strategy in the context of myocardial infarction.

## Materials and methods

### Animals

Our experiments used Wistar Han male rats (8–9 weeks, Charles River). All animals received humane care, and study protocols complied with institutional guidelines. The investigation adhered to Directive 2010/63/EU of the European Parliament and the Guide for the Care of Laboratory Animals published by the US National Institutes of Health (NIH Publication No. 85-23, revised 1996) and was approved by the local ethics committee (Comité d’Ethique de l’Expérimentation Animale Université Bourgogne Franche-Comté, Dijon, France, protocol agreement number: APAFIS#16546-2018082915228167v4). The present study is in accordance with the ARRIVE guidelines. Animals were housed at 21 ± 2 °C with a constant humidity of 55 ± 10%, following a light/dark cycle of 12 h, and had free access to water and a standard diet ad libitum.

### Assessment of GDF15 expression after in vivo ischemia reperfusion injury

#### In vivo ischemia reperfusion surgery

Rats were deeply anesthetized with isoflurane for 2 min in an induction box (2 L/min, 5% Isoflurane) and then transferred to a mask (0.6 L/min, 2% Isoflurane) and injected with buprenorphine (0.075 mg/kg). A 5% lidocaine/prilocaine (1:1) cream was applied to the incision site as a local anaesthetic (ANESDERM 5%). Once withdrawal reflex testing was achieved, animals were intubated and ventilated (VentElite 55-7040, Harvard Apparatus). A left thoracotomy was realized between the 3^rd^ and 4^th^ intercostal space. A 6.0 silk suture (K889H, Ethicon) was placed under the left anterior descending coronary artery, tightened and knotted on a catheter to interrupt blood flow and to induce 30 min of ischemia. The knot was then released and the catheter removed to initiate reperfusion. Subsequently, the rib cage was closed with a 5.0 prolene suture (EH7229H, Ethicon), and a pleural void was eliminated by aspirating excess air with a syringe. After skin closure with a 5.0 prolene suture (7475H, Ethicon), animals were awakened and placed in a 37 °C incubator until they fully recovered (Cimuka PD30SH, Ducatillon). Sham-operated animals underwent an identical procedure, the only difference being that the knot was loosely tied.

During surgery, blood samples were collected before ischemia, 30 min and 24 h after starting reperfusion from the ventral caudal artery or in the inferior vena cava depending on whether the animal was sacrificed just after. The samples were collected in tubes containing lithium heparin (15.1673, Sarstedt) and centrifuged 5 min at 5000 G to isolate the plasma. The plasma was then rapidly frozen in liquid nitrogen and stored at − 80 °C until further analysis.

After 24 h of reperfusion, rats were re-anesthetized with isoflurane and intubated following the previously described procedure. Under deep anaesthesia (isoflurane 3%), the chest was reopened, and the heart was removed. This non-painful procedure was approved by the local ethics committee. The left ventricle was divided into two distinct regions: the first contained the ischemic zone (IZ), including border zone, and the second corresponding to the non-ischemic remote zone (RZ). Tissues were promptly weighted, flash-frozen in liquid nitrogen, and stored at -80 °C until further processing.

#### Enzyme-linked immunosorbent assay (ELISA)

Circulating GDF15 concentrations were determined in thawed plasma samples using a commercial ELISA kit (MGD150, Bio-Techne) following the manufacturer’s protocol. Test specificity indicates less than 0.5% cross-reactivity with available related molecules. Briefly, samples were incubated in a microplate coated with mouse/rat monoclonal GDF15 antibody for 2 h at room temperature (RT). After multiple washes, a primary mouse/rat GDF15 antibody conjugated to horseradish peroxidase was added to each well and incubated for an additional 2 h at RT and before another wash. Colorimetric revelation was initiated by a mix of hydrogen peroxide and tetramethylbenzidine and stopped by the addition of hydrochloric acid. The optical density of each well was immediately determined using a spectrophotometer VICTOR^3^ (Perkin Elmer) at 450 nm.

#### Quantitative real-time PCR (RT-qPCR) analysis

Total RNA was extracted from frozen left rat ventricles using NucleoZOL reagent (NucleoSpin® RNA Set for NucleoZOL MACHEREY-NAGEL). RNA quality and integrity were assessed using a Nanodrop and an Agilent 2100 Bioanalyzer (G2939B, Agilent), respectively. Reverse transcription was performed using the PrimeScript RT reagent kit (RR037B, TaKaRa) following the manufacturer’s protocol. RT-qPCR was done with 2 µl of cDNA using the SYBR-Green PCR Master-Mix (Applied Biosystems) and both sense and antisense primers (5 mM) in a final volume of 20 µL, using a StepOne-Real-Time PCR system (Applied Biosystems). Data were analysed using relative quantification, normalized against GAPDH mRNA as the housekeeping gene, and presented as fold change compared to the RZ of sham-operated rats. Primers used for the amplification of rat genes are provided in Table 1.

**Table 1:** Primer sequences for RT-qPCR.

Gene	Primer	Sequence	Length (bp)
GDF15	Forward	5′-CGAGAGGACTCGAACTCAGA-3′	71
	Reverse	5′-CCCAATCTCACCTCTGGACT-3′	
GAPDH	Forward	5′-AAGGTCATCCCAGAGCTGAA-3′	138
	Reverse	5′-CTGCTTCACCACCTTCTTGA-3′	

#### Western blot analysis

Heart tissues were lysed using the Precellys® homogenizer combined with the Cryolys module (Bertin technologies) and zirconium beads (ZROB10, Next Advance) in 10 volumes of radioimmunoprecipitation assay (R0278, Sigma-Aldrich) buffer containing protease and phosphatase inhibitors (A32961, ThermoFisher) at 4 °C. Homogenates were then centrifuged at 10,000 g for 15 min at 4 °C to separate proteins from cells debris, and supernatant protein concentrations were measured using the Lowry method.

Equal protein amounts were loaded and separated on a sodium dodecylsulfate-polyacrylamide (SDS-PAGE) TGX Stain-Free FastCast gel electrophoresis (TGX Stain-Free FastCast Acrylamide kit 12%, 1610175, Bio-Rad) under reducing and denaturing conditions. Proteins were then transferred to a polyvinylidene difluoride (PVDF) membrane using Turbo Transblot technology. After blocking non-specific binding sites with 5% non-fat milk in 0.1% PBS/TWEEN 20 for 1 h at RT, membranes were incubated overnight at 4 °C with primary antibodies against anti-GDF15 (1:500, ab206414, Abcam).

Membranes were washed three times for 5 min with 0.1% PBS/TWEEN 20 and then incubated for 1 h at RT with anti-rabbit IgG HRP-linked secondary antibody (7074, Cell Signaling). Antibody reactivity was detected with WesternBright™ Sirius substrate (K-12043, Advansta) using the Chemidoc imaging system (Bio-Rad). Each revealed band was normalized by the total amount of proteins loaded on the corresponding lane obtained with the stain-free technology. Gels were run in duplicate, and chemiluminescence measurements were analysed with Image Lab software (version 6.1.0, Bio-Rad).

### Assessment of GDF15 cardioprotective effect in vivo

#### In vivo conditioning protocols

**Figure 1:**
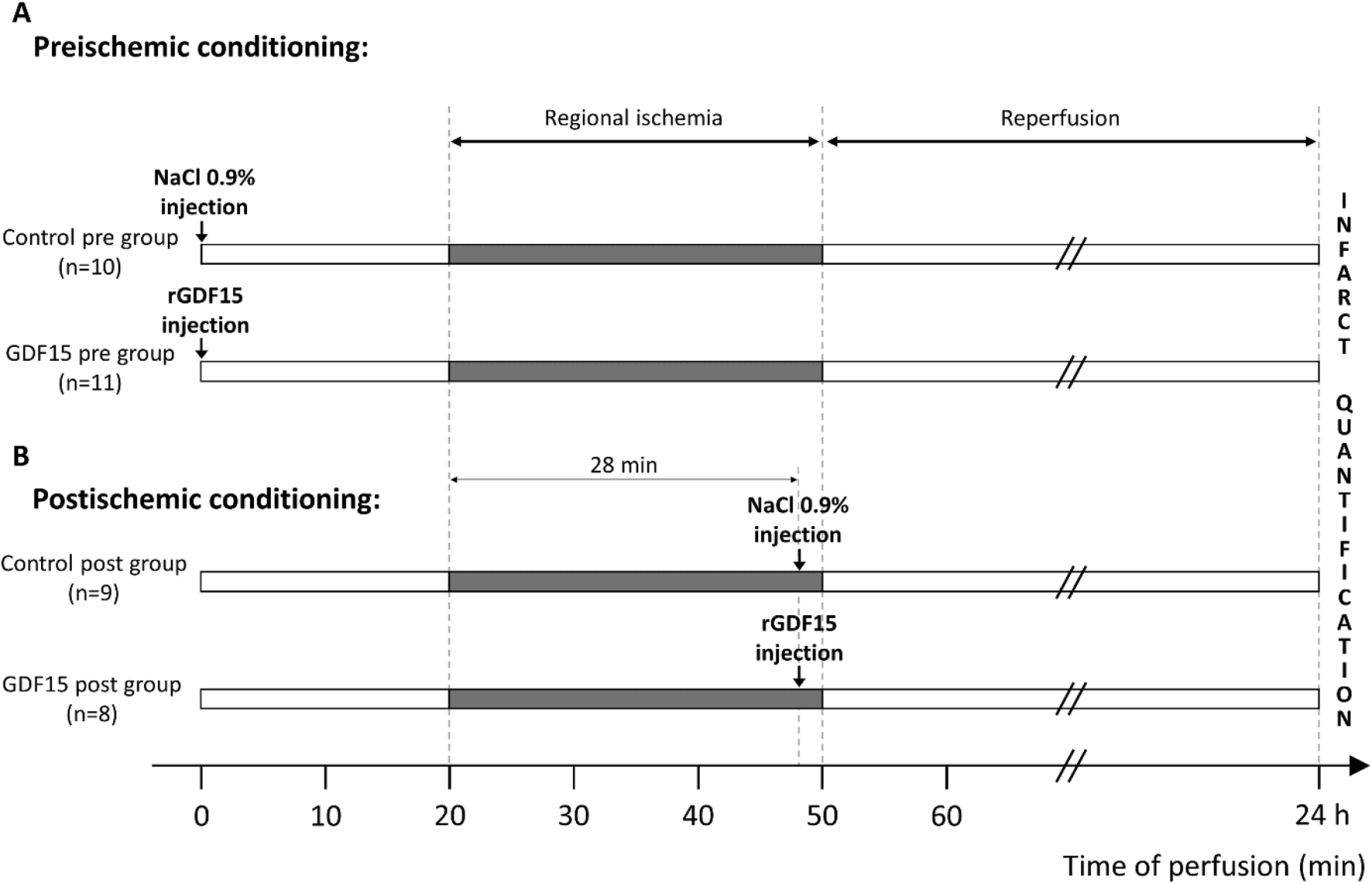
In vivo myocardial ischemia reperfusion experimental protocols. Transient regional ischemia was induced in rats by in vivo ligation of the left anterior descending (LAD) coronary artery for 30 min followed by 24 h of reperfusion. (**A**) Preconditioning protocol involved intravenous injection of saline (control) or rGDF15 (2.5 µg/kg) 20 min before ischemia; (**B**) Postconditioning protocol included intravenous injection of saline (control) or rGDF15 (2.5 µg/kg) 2 min before reperfusion (after 28 min of ischemia). Hearts were then harvested to determine the infarct size.

During surgery, rats were injected with either 0.9% saline or 2.5 µg/kg of rGDF15 in the dorsal penile vein. The injection was administered 20 min before the transient ligation of the left anterior descending coronary artery (preconditioning protocol) or after 28 min of ischemia (postconditioning protocol; Fig. 1).

#### In vivo infarct size quantification

After 24 h of reperfusion, rats were anesthetized again with isoflurane, intubated, and the chest was reopened. The cardiac suture left in place was retied to recreate the same ischemia. A 5% Evans’ blue saline solution was injected in the inferior vena cava to dye in blue all tissues except the area at risk, which was left uncoloured. Immediately afterwards, heart was harvested, and the left ventricle (LV) was isolated, slightly frozen for 15 min, and sliced into four 1 mm-thick slices from the apex to the ischemic suture. Each slice was then weighed and scanned to determine the area at risk as the percentage of the undyed area by the LV total area. Slices were incubated for 12 min in a 2% solution of 1,3,5-triphenyltetrazolium chloride (TTC, T-8877, Sigma-Aldrich) in phosphate buffer (pH = 7.4) at 37 °C, then transferred in 10% formalin for 1 h at RT. Heart slices were mounted between two microscope glass slides to scan both faces. Digital images were analysed with ImageJ software (version 1.53c, NIH). The infarct size was reported as a percentage of the area at risk.

### Evaluation of GDF15 cardioprotective effect ex vivo

#### Isolated perfused heart preparation

The perfusion medium was a modified Krebs–Henseleit bicarbonate buffer containing the following in mmol/L: 118 NaCl, 25 NaHCO_3_, 1.2 KH_2_PO_4_, 1.2 MgSO_4_·7H_2_O, 3.96 KCl, 2 CaCl_2_·2H_2_O and 5.5 glucose. It was filtered through a 0.45 µm filter (HAWP04700, Merck KGaA) to avoid any particulate contaminants and constantly gassed with 95% 0_2_ and 5% CO_2_, maintaining an adjusted end-pH of 7.4 ± 0.05 at 37 °C.

Rats were deeply anesthetized with isoflurane as described above and injected with heparin (500 IU/kg, i.p.) to prevent blood clotting. The heart was quickly removed and placed in a cold perfusion buffer bath (4 °C) until all contraction ceased. The heart was immediately cannulated by the aorta on the Langendorff system and perfused with Krebs–Henseleit perfusion buffer at a constant pressure of 80 mmHg and temperature of 37 °C. An elastic water-filled latex balloon (73–3479, Harvard Apparatus) connected to a pressure transducer was introduced into the LV to measure intra-ventricular pressures. The balloon was inflated to obtain an initial left ventricular end-diastolic pressure (LVDP) of 8–13 mmHg and left unchanged. The following functional parameters were recorded: heart rate (HR), left ventricular end-systolic pressure (LVSP), LVDP, left ventricular developed pressure (LVDevP = LVSP − LVDP) and the first derivative of the LVDevP: the left ventricular maximal pressure development (+ dP/dt) and left ventricular minimal pressure development (− dP/dt) (PowerLab®, LabChart® System, ADInstruments). Coronary flow (CF) was measured by time-point collection of the effluent.

#### Ex vivo conditioning protocols

**Figure 2:**
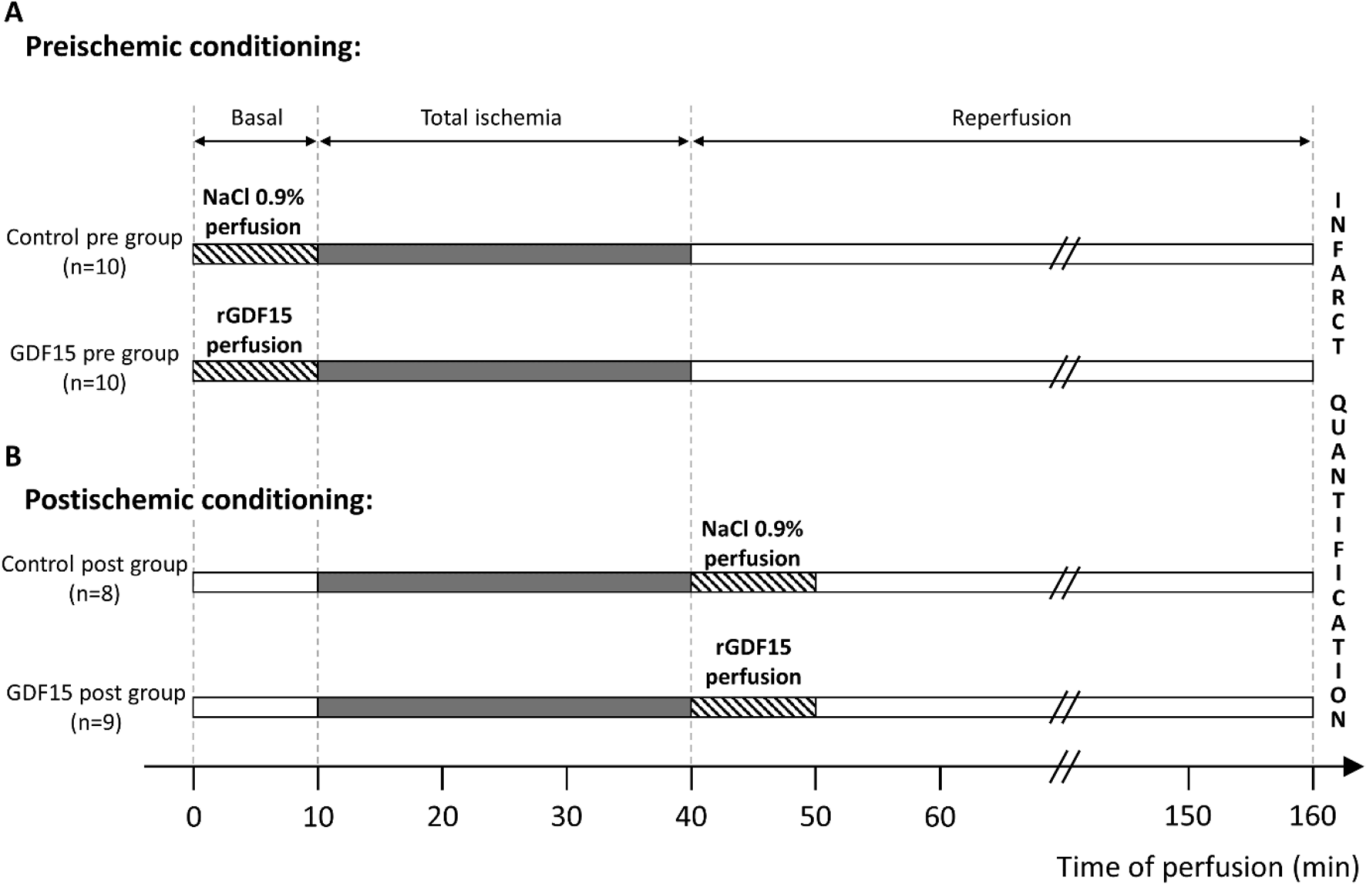
Ex vivo myocardial ischemia reperfusion experimental protocols. Transient global ischemia was induced by stopping the supply of Krebs–Henseleit buffer in isolated perfused rat hearts for 30 min, followed by 2 h of reperfusion. (**A**) Preconditioning protocol: saline (control) or rGDF15 (2 µg/L) were perfused 10 min before ischemia; (**B**) Postconditioning protocol: saline (control) or rGDF15 (2 µg/L) were perfused during the initial 10 min of reperfusion. Functional parameters (coronary flow, left ventricular pressure, heart rate) were measured throughout the experiment. At the end of the procedure, the cardiac infarct size was determined.

Hearts were allowed to stabilize for approximately 10–15 min before basal cardiac parameters were recorded. During the 10 min of basal measurements, isolated hearts were perfused with either 4 mM HCl (as control perfusion, rGDF15 diluent) or 2 µg/L rGDF15 (8944-GD, Bio-Techne) in the preconditioning protocol. Alternatively, in the postconditioning protocol, perfusion was done during the first 10 min of reperfusion. In both protocols, control and rGDF15 perfusion were adjusted to 1/100th of the coronary flow (Fig. 2).

#### Ex vivo infarct size quantification

At the end of the 2-h reperfusion period, hearts were removed from the Langendorff system, promptly dried, weighted, and briefly frozen at − 20 °C for 15 min to harden the tissue before slicing. Subsequently, the total ventricular area was divided into 6 transverse sections of 1 mm in thickness, and incubated with TTC to evaluate infarct size, as described above. The infarct area was calculated as the percentage of the total ventricular area.

### Statistical analysis

Data sets were analysed using SigmaPlot (version 12.5, Systat Software), and statistical significance was set at *P* < 0.05. Normality and homoscedasticity of data sets were verified before applying parametric tests, and differences between two groups were thus assessed with a Student’s t-test. GDF15 pharmacokinetics were assessed using a one-way ANOVA for repeated measures, and ex vivo heart function using a two-way ANOVA for repeated measures, followed by a Tukey’s post hoc analysis, as indicated in each figure. Data are represented as mean ± SD. The circulating levels of GDF15, the area at risk, and the infarct size are represented as box plots, showing the median value and interquartile ranges (25th and 75th percentile).

## Results

### GDF15 is expressed at the gene and protein level in the ischemic zone after in vivo myocardial infarction

**Figure 3:**
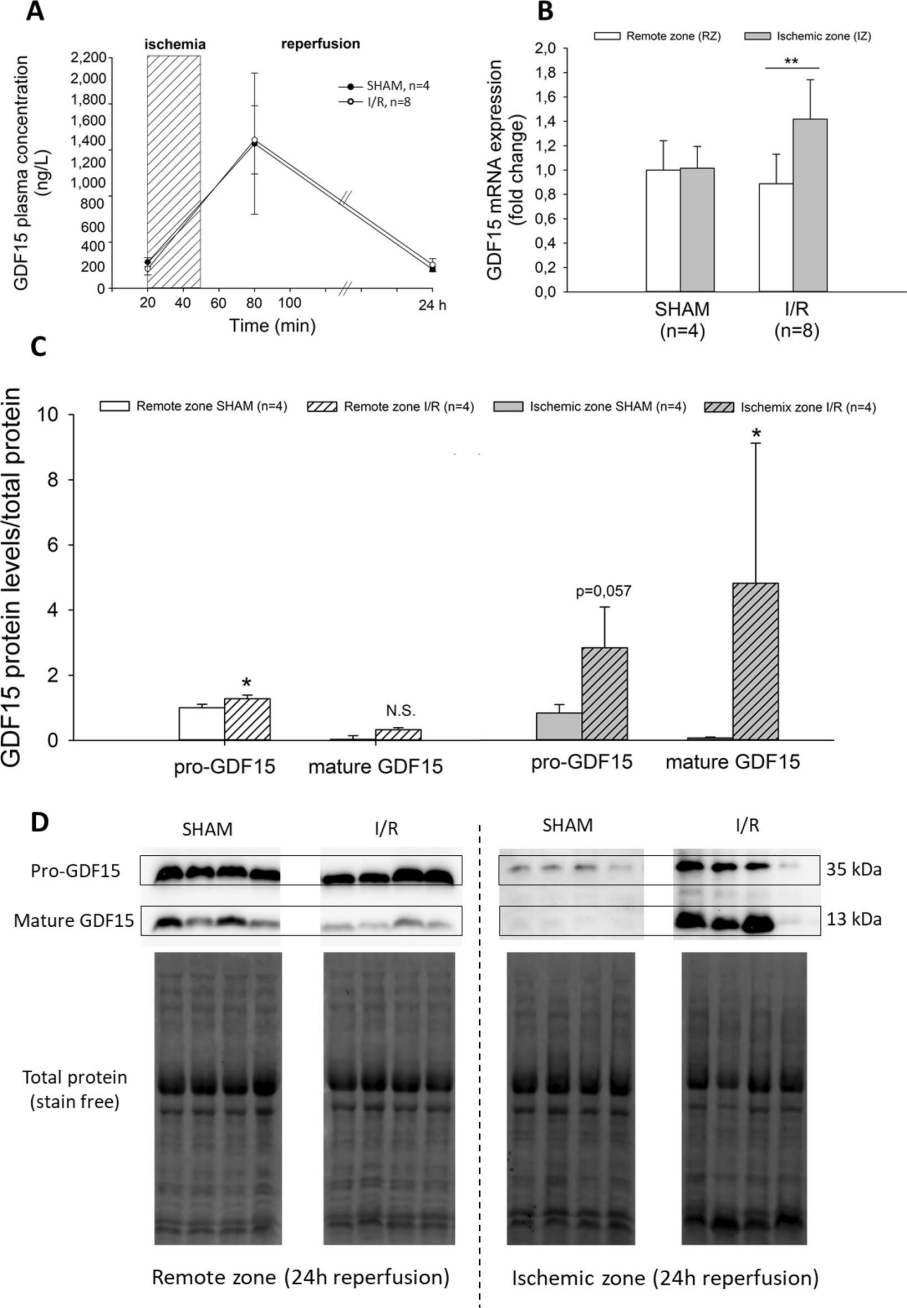
GDF15 circulating levels and cardiac expression in response to myocardial I/R or sham operation in rats. (**A**) GDF15 plasma levels before ischemia, after 30 min and 24 h of reperfusion; (**B**) GDF15 mRNA expression in the remote and the ischemic zone of the ischemic heart after 24 h of reperfusion. **P < 0.01 vs remote zone using ANOVA; (**C**) Pro- and mature GDF15 protein expression in the remote and the ischemic zone of the ischemic heart after 24 h of reperfusion. *P < 0.05 vs sham using Student test; (**D**) Pro- and mature GDF15 chemiluminescence from the total protein blotted.

During surgery, preischemic GDF15 circulating levels were around 200 ng/L in both groups of rats: 283 ± 24 ng/L in the sham group and 211 ± 24 ng/L in the I/R group (Fig. 3A). Thirty minutes after starting reperfusion, GDF15 peaked at around 1,600 ng/L in both I/R and sham-operated rats (1568 ± 383 ng/L in sham, 1609 ± 131 ng/L in I/R). 24 h after reperfusion, GDF15 levels returned to their preischemic levels in both groups (205 ± 13 ng/L in sham, 255 ± 24 ng/L in I/R).

In hearts collected 24 h after reperfusion, there was an increase in GDF15 mRNA expression observed in the IZ of hearts from I/R rats, but not in those from sham-operated rats (Fig. 3B). Additionally, in I/R hearts, there was an increase in pro-GDF15 expression in both the RZ and the IZ, while in the IZ, a six-fold increase in mature GDF15 expression was observed (Fig. 3C, D, see supplemental data for full-length gels) .

### rGDF15 preconditioning, not postconditioning, improves myocardial I/R injury

**Figure 4:**
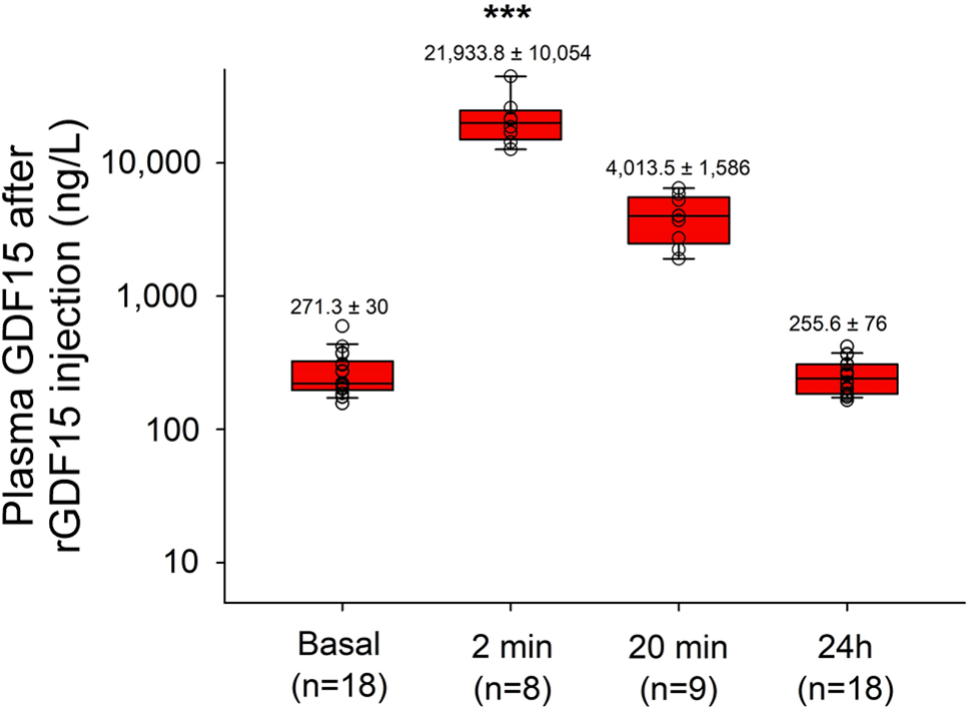
Plasma levels of GDF15 after intravenous injection of 2.5 µg/kg rGDF-15. ***P < 0.001 vs. basal levels using one-way ANOVA for repeated measures followed by Holm-Sidak’s post-hoc analysis.

In order to assess whether GDF15 exerts cardioprotective effects during in vivo myocardial I/R injury, recombinant GDF15 (rGDF15) was administrated intravenously before or after post-ischemic reperfusion. Our objective was to achieve GDF15 concentrations relevant to those observed in patients with a high CV risk, i.e. 2000–4000 ng/L^34,35^. For this purpose, we conducted pharmacokinetic assays in anesthetized rats intravenously injected with 2.5 µg/mL rGDF15. Baseline GDF15 circulating levels were 271 ± 26 ng/L (Fig. 4). Immediately after rGDF15 injection, GDF15 plasma levels increased to levels above 20,000 ng/L. However, 20 min after injection, GDF15 levels reached 4013 ± 528 ng/L in the GDF15 group while remaining stable at 211 ± 23 ng/L in the control group. 24 h after rGDF15 injection, control and GDF15 groups returned to similar baseline levels around 204 ± 13 and 233 ± 24 ng/L, respectively (Fig. 4).

**Figure 5:**
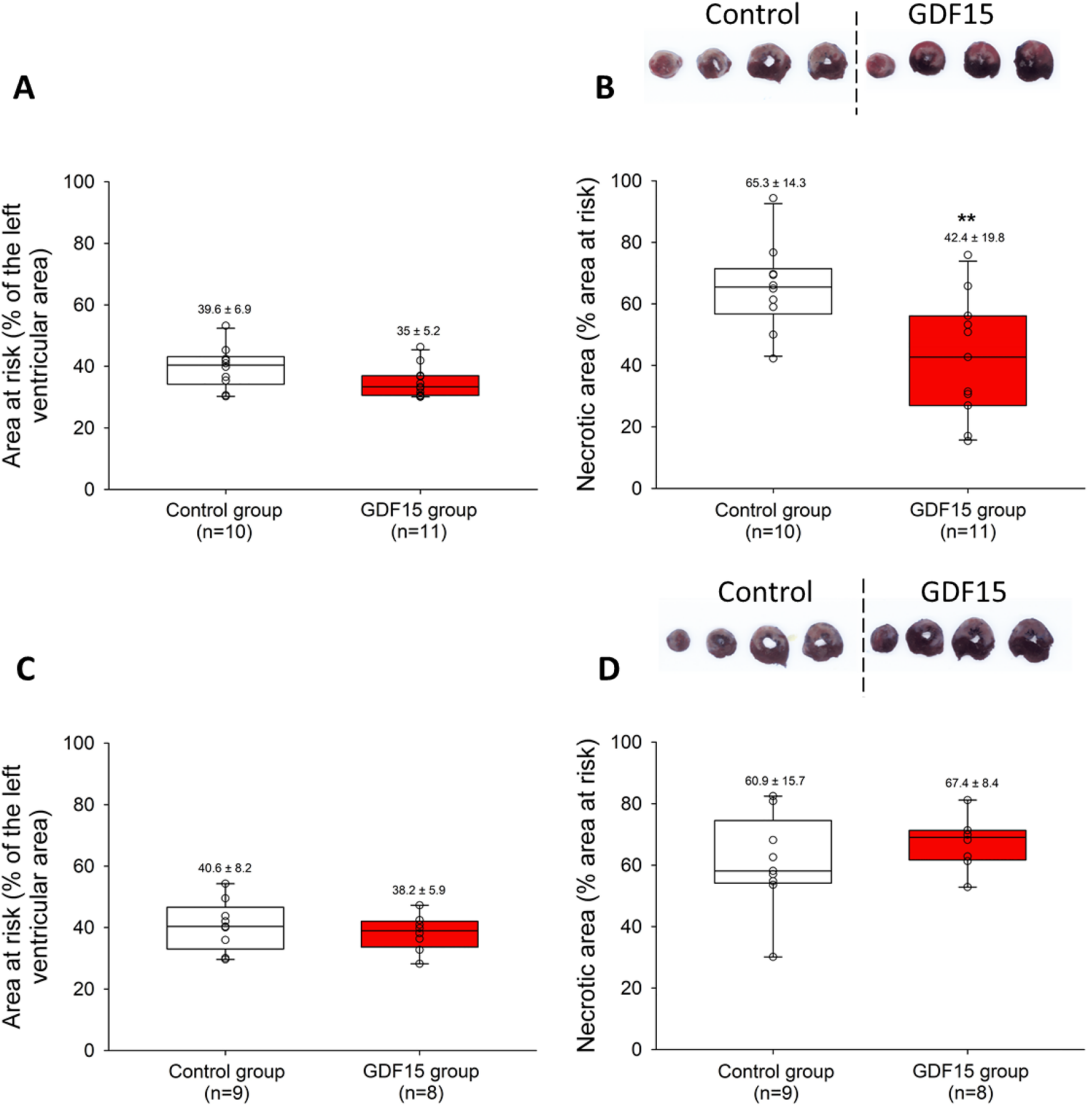
Area at risk (**A**, **C**) and infarct size including representative heart slices stained with Evans’s blue and TTC from one control and one rGDF15-treated rat (**B**, **D**) assessment after 24 h of reperfusion following 30 min of left anterior descending coronary artery ligation in rats intravenously injected with saline (control) or rGDF15 (2.5 µg/kg) either (**A**, **B**) 20 min before ischemia: preconditioning protocol; or (**C**, **D**) 2 min before reperfusion: post conditioning protocol. **P < 0.01 vs control group using Student test.

The cardioprotective effects of rGDF15 in both pre- and postconditioning protocols were evaluated in vivo, as described above. 24 h after ischemia, the AAR, determined by Evans’ blue dye injection, was similar in the control and GDF15 groups in both pre- and postconditioning protocol groups (Fig. 5A and C). Consequently, infarct sizes could be rigorously compared between the groups. In the preischemic protocol, rGDF15 reduced infarct size (65 ± 5% in the control group vs. 42 ± 6% in the GDF15 group, *p* < 0.01, Fig. 5B). However, in the postconditioning protocol, both experimental groups displayed a similar necrotic area (61 ± 5% of the AAR in the control group vs. 67 ± 3% in the GDF15 group, Fig. 5D).

### rGDF15 preconditioning, not postconditioning, improves ex vivo heart recovery after I/R

**Figure 6:**
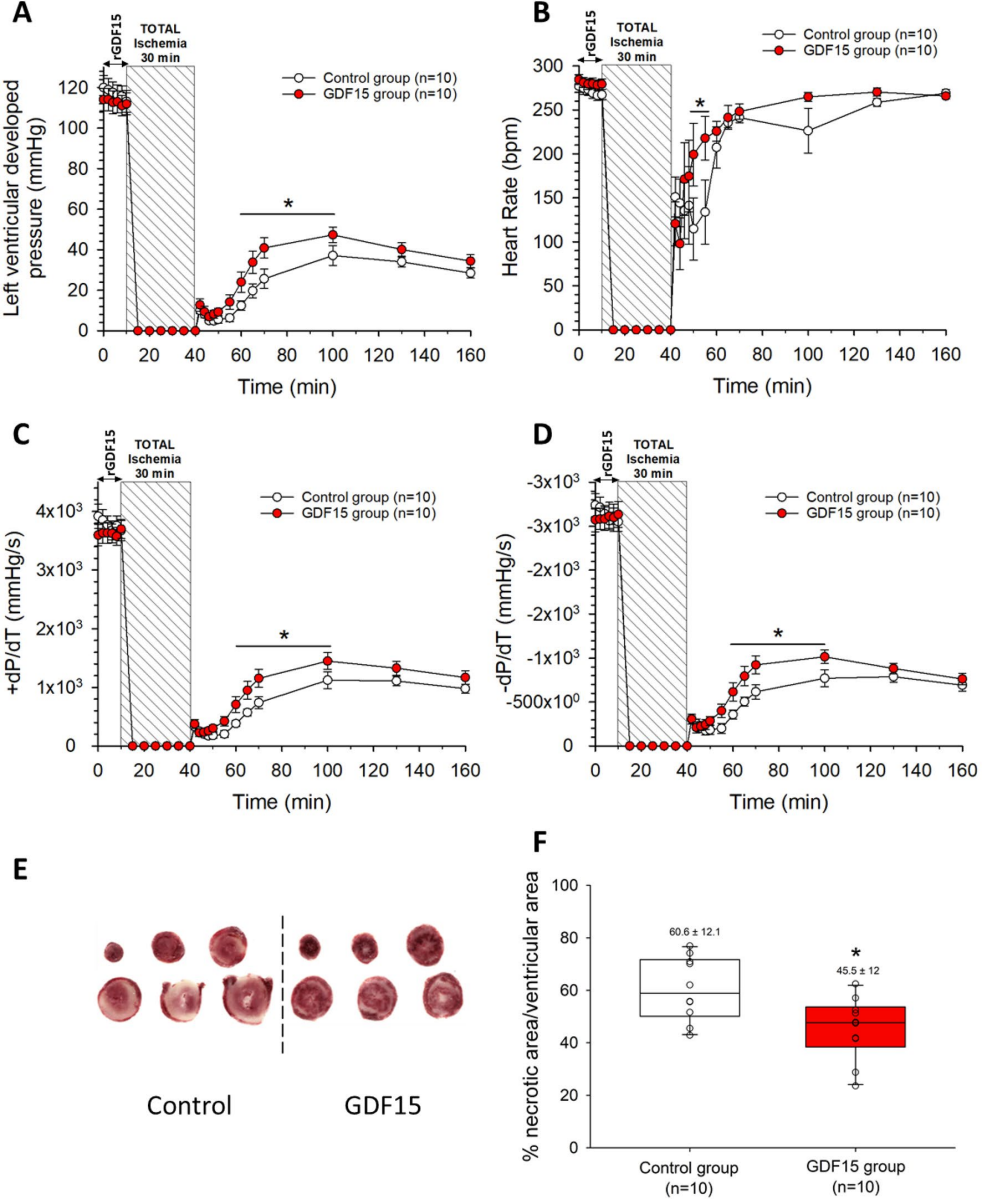
Time course of contractile parameters of (**A**) LVDevP; (**B**) Heart rate (**C**) + dP/dt; (**D**) − dP/dt during the ex vivo control and rGDF15 preconditioning protocol; (**E**) Representative heart slices stained with TTC from one control and one rGDF15-treated heart; (**F**) Infarct size at the end of the 2-h reperfusion. *P < 0.05 vs control group using two-way ANOVA for repeated measures with Tukey’s post hoc analysis.

To consolidate our findings, we extended our protocol to an ex vivo model to assess the potential protective effect of rGDF-15 independently of neural, humoral or inflammatory response regulators. During the basal period, where the isolated hearts were perfused with or without rGDF15, all measured parameters were similar between the two groups (Fig. 6). When perfusion was stopped to induce ischemia, hearts rapidly stopped beating, decreasing their contractile parameters to zero (HR, LVDevP and ± dP/dt, Fig. 6A, B, C, and D). At the onset of reperfusion, isolated hearts exhibited a slight transient peak and drop-off before gradually recovering their contractile functions over time. The recovery was only partial in both groups, reaching 30–40% of their initial LVDevP and ± dP/dt, with maximum recovery at 60 min of reperfusion, followed by a gradual decline due to the limitations of the ex vivo model (Fig. 6A). However, hearts perfused with rGDF15 prior to total ischemia demonstrated better recovery of LVDevP, + dP/dt and − dP/dt during the early reperfusion period. Hearts perfused with rGDF15 displayed an improved recovery of HR during early reperfusion, attributed to fewer episodes of arrythmia and a better return to sinus rhythm (Fig. 6B). At the end of the 2-h reperfusion, TTC staining of heart slices (Fig. 6E) revealed a greater infarct size in the control group than in the rGDF15 group: 60 ± 4 and 45 ± 4% of the LV, respectively (Fig. 6F).

**Figure 7:**
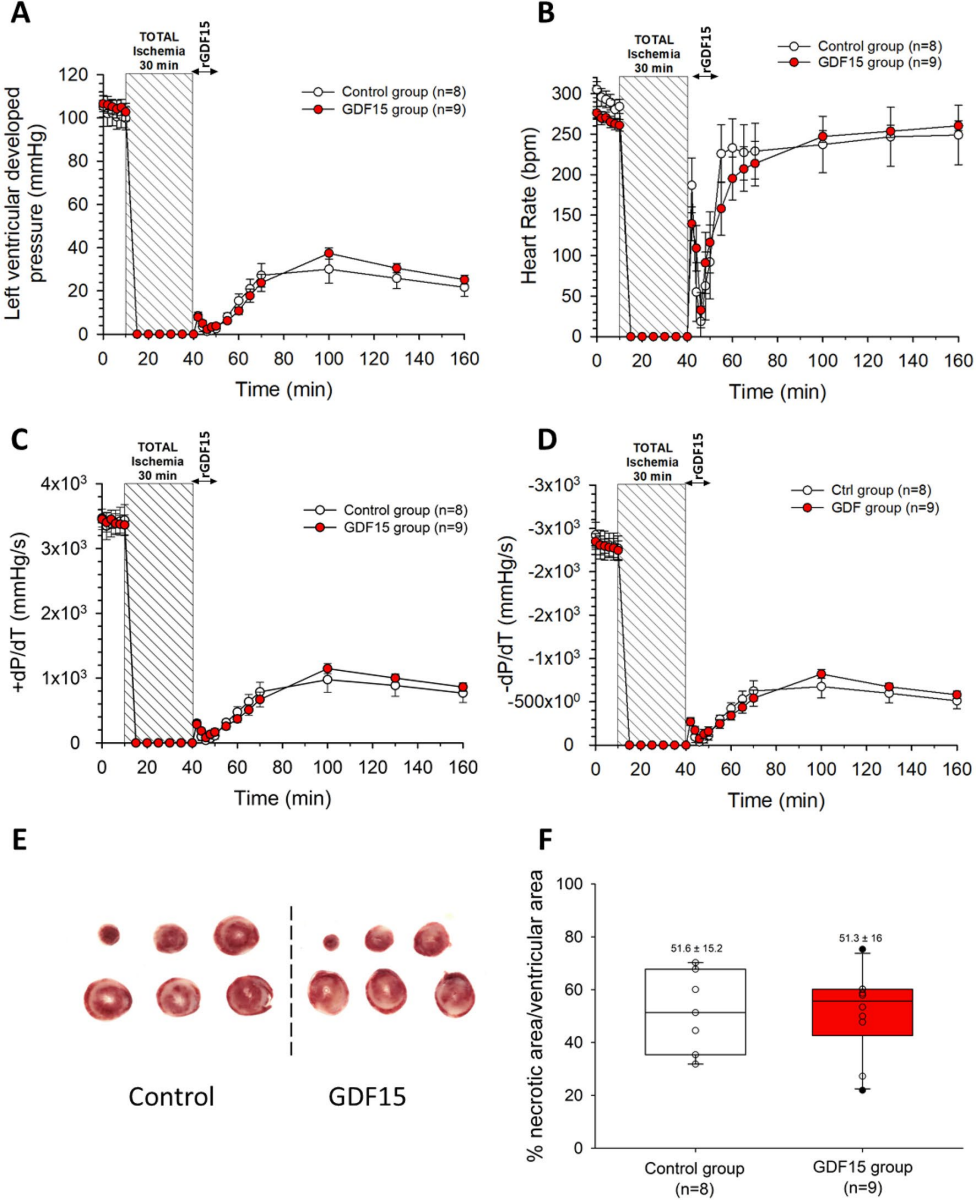
Time course of (**A**) LVDevP; (**B**) Heart rate; (**C**) + dP/dt; (**D**) − dP/dt of isolated perfused hearts during the ex vivo control and rGDF15 post-conditioning protocol (**E**) Representative heart slices stained with TTC from one control and one rGDF15-treated heart; (**F**) Infarct size at the end of the 2-h reperfusion.

We also assessed the ex vivo post-ischemic effects of rGDF15 by perfusing it during the first 10 min of reperfusion. During the basal period, all measured parameters (LVDevP, ± dP/dt and HR) were comparable between the two groups (Fig. 7A, B, C, and D). Following 30 min of total ischemia, we observed a similar pattern of recovery in functional parameters as described in our previous set of ex vivo experiments (Fig. 7A, B, C and D). However, no significant differences were identified between the control and GDF15 groups in any of the monitored parameters. At the end of reperfusion, the size of the infarct (Fig. 7E) was 52 ± 6% of the total ventricular area in the control group, similar to the GDF15 group in which the infarct size was 51 ± 5% of the total ventricular area (Fig. 7F).

## Discussion

The present study found GDF15 to be a cardioprotective cytokine induced in response to myocardial I/R injury, in line with previous findings^25,30^. Our work demonstrates the expression of GDF15 in the heart tissue following in vivo I/R injury and suggests a potential role for rGDF15 as a therapeutic molecule. Surprisingly, the elevation of plasma GDF15 during reperfusion did not differ between I/R and sham-operated rats. To our knowledge, there is currently no in vivo data available on systemic GDF15 secretion in response to cardiac I/R surgery. However, this aligns with the stress-sensor role of GDF15, secreted into the circulation in response to injuries, cellular damage, oxidative stress and inflammation^14^, which are common elements induced by cardiac surgery. The origin of circulating GDF15 in this situation remains a matter of debate, but it is possible that the vago-splenic axis, which is activated during remote ischemic conditioning^36^, may play a role. GDF15 was found to be expressed in erythroblasts^37^, suggesting that the spleen might serve as a release organ for GDF15 in response to neurohumoral activation of the sympathetic nervous system. It has been proposed that catecholamines, released during acute myocardial or cerebral ischemia, may remotely induce GDF15 secretion^28^. In a recent study, local anaesthetics such as lidocaine were shown to upregulate GDF15 production in HeLa cultured cells^38^, suggesting that lidocaine use in our surgical procedure may have contributed to the GDF15 secretion observed in both I/R and sham rats. Additionally, two studies indicate that coronary artery bypass grafting in patients increases GDF15 plasma level by 2.5- or 3-fold compared to anaesthesia induction level^39,40^. These findings suggest that thoracic surgery itself induces GDF15 secretion. In our model, the initial plasma level of GDF15 was very low (≈ 200 ng/L), which can be explained by the young age and good health of the rats. However, we observed a 10-fold GDF15 increase after 30 min of I/R followed by 30 min of reperfusion. Patients from clinical studies often have stable coronary artery disease or a history of other cardiovascular diseases. Hence, their GDF15 level is already elevated at baseline (≈ 1000 ng/L)^22^, and increased further after thoracic surgery^39^, but to a lesser extent than what is observed in rats.

We found that I/R upregulates GDF15 expression in the ischemic part of the heart compared to the RZ after 24 h of reperfusion, while no differences were observable in sham-operated rats. This finding is consistent with observations by Zhang and Kempf in an I/R mouse model, where GDF15 mRNA was exclusively upregulated in the infarcted area after 1 h of ischemia, reaching a transcriptional peak at 24 h of reperfusion^25,41^. Interestingly, Kempf et al. also demonstrated that only a permanent ligation of the left anterior descending coronary artery induced GDF15 mRNA upregulation in the RZ, suggesting that GDF15 may be expressed remotely when stress signals reach a certain level. At the protein level, I/R rats exhibited an increase in pro-GDF15 in the RZ and a strong trend in the IZ compared to sham rats after 24 h of reperfusion. These results align with expression kinetics reported by other authors, showing an increase in pro-GDF15 in the ischemic heart compared to sham mice after 4 h of reperfusion, peaking at 24 h. In vitro studies have also demonstrated that cardiomyocytes in culture produce pro-GDF15 in simulated I/R, in both supernatant and cell lysate^25^. Interestingly, mature GDF15 was only detected in supernatants after either 6 h of hypoxia or 3 h of hypoxia followed by 3 h of reoxygenation in vitro, suggesting rapid and efficient secretion into the circulation in response to in vivo I/R. Our work describes, for the first time, a cardiac expression of mature GDF15 in response to in vivo I/R. I/R hearts exhibited a significant six-fold increase in mature GDF15 in the IZ 24 h after the onset of reperfusion, but no difference in the RZ. Additionally, while our results corroborate that GDF15 is upregulated in the heart after ischemia^25^, it is important to consider that, in addition to cardiomyocytes, cardiac resident immune cells may also serve as a source of GDF15.

The presence of biologically active mature GDF15 after I/R suggests that it has a cardioprotective and anti-hypertrophic role, consistent with previous findings^25^. GDF15’s cardioprotective properties involve both direct anti-apoptotic effects and inhibition of pro-inflammatory leukocyte recruitment in the infarcted area^32,41,42^. GDF15-deficient mice subjected to ischemia displayed increased leukocyte recruitment in the infarcted zone, leading to higher mortality and a greater infarct size with more apoptotic cardiomyocytes. Conversely, GDF15 over-expression in mice reduced I/R injury by lowering the number of apoptotic cells, reducing neutrophil infiltration, and decreasing pro-inflammatory cytokine expression in the heart^31^. In our in vivo and ex vivo models, preischemic rGDF15 administration also reduced the infarct size, suggesting a direct cardioprotective effect of GDF15. Our data suggest a cardiac-specific effect of GDF15, potentially interacting with distinct receptor from GFRAL, which is not expressed in the heart, or indirectly via other mediators. Unfortunately, studies exploring GDF15 pathways in cardiomyocytes in vitro have mainly used commercial human rGDF15 (rhGDF15), which may be contaminated by TGF-β, as reported by several authors^43–45^. Olsen et al. demonstrated that some rhGDF15 batches were activating TGF-β receptors and their canonical Smad pathway. Moreover, TGF-β signalling also includes non-canonical signalling proteins like ERK, Akt and mTOR, all implicated in cardioprotective processes^46,47^ and activated by rhGDF15 in vitro. Due to this contamination issue, the results from studies potentially using contaminated rhGDF15 have been called into question. As a precaution, we will not cite these experiments. However, it is important to note that batches of rGDF15 amplified in TGF-β-deprived hosts, such as *E. coli*, do not exhibit this contamination. This consideration motivated our choice to use this type of recombinant protein.

Apart from regulating leukocyte recruitment, GDF15 may exert direct intracellular cardioprotective mechanisms. Kempf et al. demonstrated that pre-treatment of cardiomyocytes with recombinant GDF15 reduced necrotic and apoptotic cell death via PI3K and Akt-dependent mechanisms^25^. A study using GDF15-overexpressing mice in a cold I/R heart grafting context reported that GDF15 promoted Foxo3a phosphorylation (p-Foxo3a) through PI3K/Akt activation and NF-κB inhibition^31^. These two kinases are members of the reperfusion injury salvage kinase (RISK), one the two major cardioprotective pathways known to limit reperfusion injuries and cell death by inhibiting mitochondrial transition pore opening (mPTP)^48^. Foxo3a is a transcription factor that regulates the cell cycle, autophagy and pro-apoptotic genes, and its translocation to the cytoplasm upon phosphorylation promotes cell survival after renal, cerebral and cardiac I/R^49–52^. Moreover, NF-κB is a complex pro-inflammatory transcription factor, and specific cardiac inhibition of NF-κB has shown cardioprotective effects in ex vivo and in vivo I/R models^53,54^.

Finally, we reported that rGDF15 failed to protect the heart when administered at the onset of reperfusion both in vivo and ex vivo, despite its beneficial effect when used before ischemia as a preconditioning compound. Pharmacological cardioprotection is challenging because it must selectively address cardiomyocyte dysfunction induced by ischemia and reperfusion without adversely affecting other physiological processes^55^. The three main pathways for achieving this, the survivor activating factor enhancement (SAFE) pathway, the RISK pathway and the NO/PKG pathway, all ultimately lead to mitochondrial protection and survival^56^. For instance, volatile anaesthetics (isoflurane, sevoflurane and desflurane) are known to be cardioprotective molecules both in pre- and postconditioning, capable of activating the RISK, SAFE and NO pathways^56^. However, one study reported that isoflurane induced a different gene expression profile depending on whether it was used as pre- or postconditioning after ex vivo myocardial I/R^57^. Nevertheless, cardioprotective molecules can also have interactions. Desflurane and propofol have been reported to be cardioprotective individually, but their combination abolished their effectiveness in cardiac conditioning^58^. We used isoflurane during the surgery, so interaction between isoflurane and our protocol for GDF15 administration during myocardial I/R can be hypothesized. Finally, preconditioning can be seen as a mechanism that delays the development of infarct size, while postconditioning actually decreases the infarct size^55^. Furthermore, the spatiotemporal organization of the RISK, SAFE and NO/PKG pathways is not completely elucidated, resulting in disparities between pre- and postconditioning^59^. This is why the most promising pharmacological cardioprotective molecules should activate several protective pathways, ensuring mitochondrial protection via redundant pathways and enabling better and broader cardioprotection^56,60,61^. It is also worth noting that the exploration of the cardioprotective abilities of GDF15 in our animal models is limited by the usage of isoflurane, a well-known cardioprotective molecule. However, data from our laboratory showed that isoflurane cardioprotection on the ex vivo Langendorff model was abolished by 30 min of ischemia, motivating our choice to use the same duration (data not shown). Further studies are warranted to reinforce our findings regarding the beneficial cardioprotective effect of rGDF15 administration in the setting of myocardial ischemia–reperfusion and to elucidate the underlying mechanisms involved. As recommended by the IMPACT criteria guidelines^62^, it is advisable to undertake long-term studies with mortality and heart failure endpoints, replicate the study in aged mice or mice with cardiovascular risk factors, validate the findings with other research groups, and extend the investigation to larger animal models. Finally, it would be interesting to see whether rGDF15 enhances cardioprotection when combined with other conditioning strategies. This would position rGDF15 as a promising candidate for a multi-target approach to enhance cardioprotection^25,63^.

## Conclusions

Our study underscores the myocardial production of GDF15 during ischemia–reperfusion and reveals its potential ability to elicit preischemic cardioprotection in both in vivo and ex vivo settings. Intriguingly, these findings suggest mechanisms that operate independently of the immune, endocrine, or nervous systems. On the contrary, administering recombinant GDF15 (rGDF15) at reperfusion failed to confer beneficial effects, implying its limited capacity to counteract the detrimental changes that occur during ischemia. However, the cardioprotective potential of GDF15 may find application in other situations, such as conditions involving the stroke-heart syndrome.

While our study sheds light on the preischemic cardioprotective effects of GDF15, the specific pathways underlying its protective mechanisms remain largely unexplored. A crucial step for future research may involve investigating its potential to protect the mitochondria, which would offer a more comprehensive understanding of its beneficial capacities. This knowledge could pave the way for therapeutic strategies, especially in the context of cardiovascular diseases and related complications.

### Supplementary Information

Supplementary Information.

## Data Availability

The data and support of these findings is available through contacting the corresponding author.
