# Supplementary figures and images for: Growth/differentiation factor 15 (GDF15) expression in the heart after myocardial infarction and cardioprotective effect of pre-ischemic rGDF15 administration
Source: Sci Rep. 2024 Jun 5;14:12949. doi: 10.1038/s41598-024-63880-5 (PMC11153639; doi:10.1038/s41598-024-63880-5)

Remote zone of the heart

Repeat 1

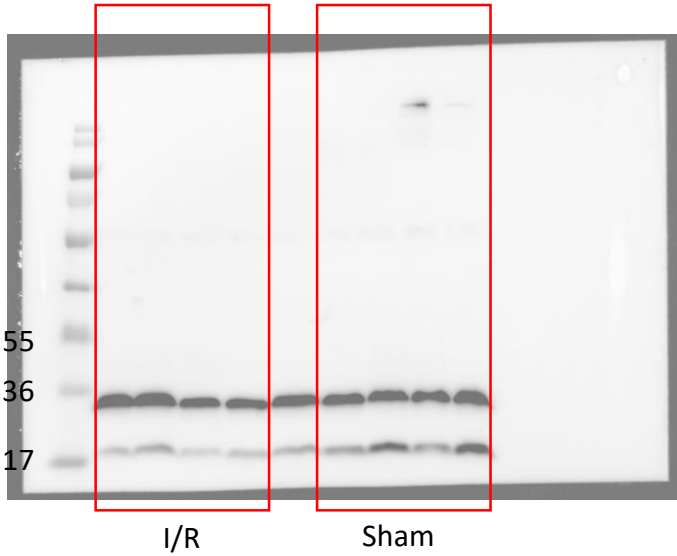

Repeat 2

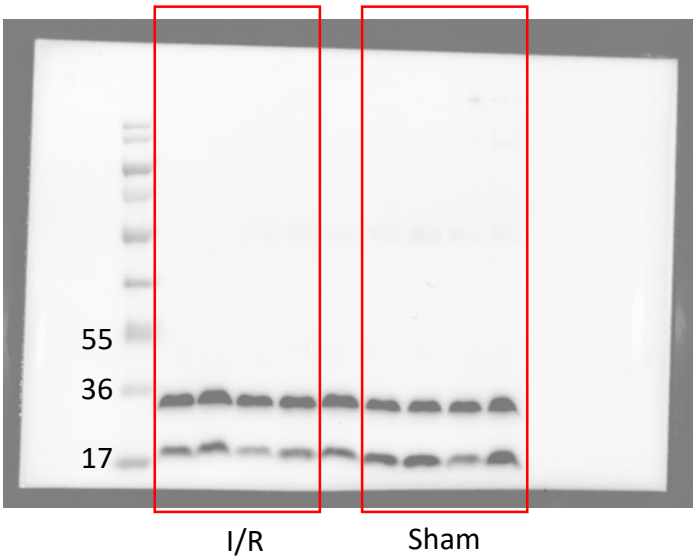

Ischemic zone of the heart

Repeat 1

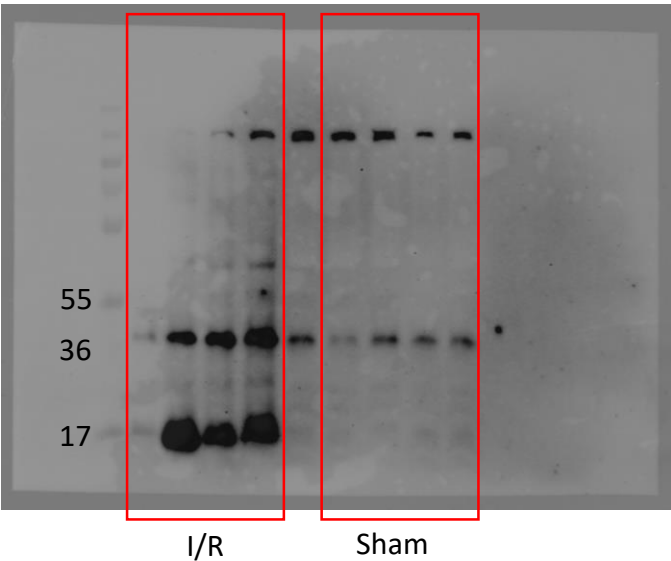

Repeat 2

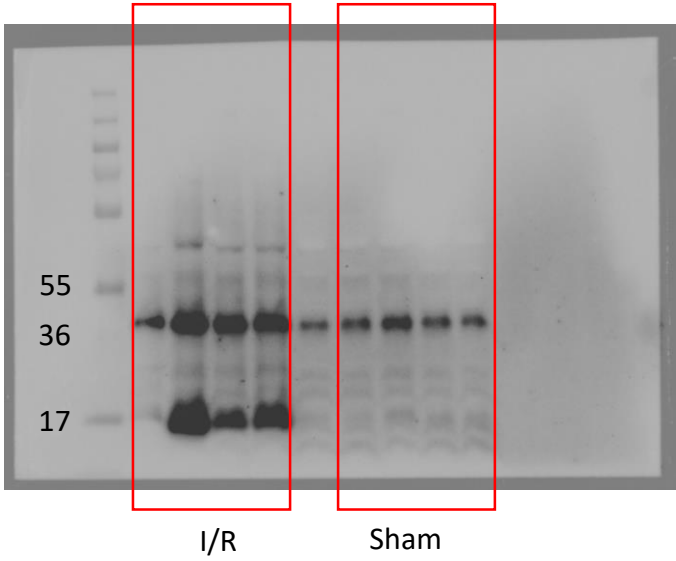

Supplement: Supplementary file 1 — Supplementary Information. [file 41598_2024_63880_MOESM1_ESM.pdf]
